# Depression, anxiety and stress among metastatic breast cancer patients on chemotherapy in China

**DOI:** 10.1186/s12912-023-01184-1

**Published:** 2023-02-06

**Authors:** Yi-Qiang Guo, Qing-Mei Ju, Miaoning You, Yang Liu, Azlina Yusuf, Lean Keng Soon

**Affiliations:** 1 School of Nursing, Capital Medical University, Beijing, China; 2 Emergency General Hospital, Beijing, China; 3 Key Laboratory of Carcinogenesis and Translational Research (Ministry of Education/Beijing), Department of Breast Oncology, Peking University Cancer Hospital & Institute, Beijing, China; 4 School of Medicine, Xia Men University, Fujian, China; 5 School of Health Sciences, Universiti Sains Malaysia, Kubang Kerian, Kelantan Malaysia

**Keywords:** Metastatic breast cancer, Depression, Anxiety, Psychological stress, Coping, Chinese

## Abstract

**Objective:**

This study aimed to assess the level of depression, anxiety and stress among metastatic breast cancer (MBC) patients undergoing chemotherapy (CT) in Beijing, China.

**Methods:**

A cross-sectional study was conducted on 176 MBC women receiving CT, selected by purposive sampling. Data were collected using self-administered questionnaires that included participants’ socio-demographic status, DASS-21 and Brief COPE. Data were analyzed using descriptive statistics and general linear regression analysis.

**Results:**

The incidence of depression, anxiety and stress among MBC women were 52.3%, 60.2% and 36.9%, respectively. General linear regression showed that age, marital status, monthly income, physical functioning, emotional functioning, pain, dyspnea, and appetite loss were associated with depression. All variance determined the depression (R^2^) was 35.6%. Marital status, self-blame and behavioral disengagement were the predictors of stress and accounted for a 35.4% stress variance in MBC women.

**Conclusion:**

Our study demonstrated depression, anxiety, and stress prevalence are high in MBC women. Assessment of psychological distress (depression, anxiety, and stress) is important to recognise MBC patients who need help and further medical and mental help support. This study’s findings can increasingly highlight that depression, anxiety, and stress are substantial problems in MBC patients. Therefore, psychological interventions are needed to reduce depression, anxiety, and stress for MBC patients.

## Introduction

Globally, breast cancer (BC) is the most common cancer and death in women and has a mortality-to-incidence ratio of 15% [1, 2]. In China, BC accounts for 19.9% of new cases [3] and is expected to affect 1.02 million women during the next five years [4, 5]. Despite the significant advancement in the comprehensive treatment of BC, the metastatic illness usually appears after a long time of undetectable disease following surgery or systemic therapy due to relapse or recurrence [6]. Although the median survival time for MBC women is three years, the overall range is more extensive because MBC survivors carry the illness for an extended period [7]. As a result, the population of MBC survivors who would have to go through a protracted anti-cancer journey is rising. Because advanced BC is so complex, it can be a single site, a single nidus, numerous sites, or multiple niduses. Chemotherapy (CT), endocrine therapy, targeted therapy, immunotherapy, and topical cancer treatment are examples of systemic cancer treatment techniques (radiotherapy, surgery, radiofrequency ablation and interventional treatment). Patients can benefit from these therapies, allowing them to live longer lives. Nonetheless, chemotherapy (CT) remains the most often used treatment [8].

Living with terminal metastatic cancer and receiving constant therapy can cause psychological discomfort and maladaptive coping. MBC patients encounter unfavourable medical, psychological, and social consequences of cancer treatment, in addition to the shock, dread, anxiety, and uncontrollable feelings of uncertainty and mortality accompanying the MBC diagnosis. As a result, MBC women experienced high psychological anguish [7, 9, 10]. Previous research has revealed various coping methods for MBC women to face their life-threatening illness and long and difficult treatment, including decision-making. However, emotional support was the most commonly used coping strategy among newly diagnosed incurable cancer patients [11, 12]. When diagnosed with a terminal illness, denial coping is frequent. It can be beneficial or harmful in managing the health situation. Individuals can adjust to the situation by using denial coping as the first step in dealing with a life-threatening disease. However, when denial coping persists and hinders persons from adapting to the truth and obtaining therapy, it can become a problem [13]. The level of social support determines the types of coping techniques used by women with BC [14]. Problem-based coping, emotion-based coping, and maladjustment were too broad to represent the coping strategies of MBC patients [15].

Cancer survivors have trouble continuing or returning to work following primary treatment, such as CT and radiation therapy [16]. However, there has been little emphasis on MBC women’s psychological distress and coping styles, particularly in mainland China. Although the association between coping styles and psychological suffering has been extensively researched, the classification of coping styles (e.g. engagement coping/disengagement coping; functional coping/ dysfunctional coping) is rather broad. It is well-known that metastasis cancer and its treatment have a debilitating effect on patients and coping strategies were a prerequisite in the cancer-fighting process. In addition, the disease are likely to affect the mental wellbeing of MBC women, potentially contributing to depression, anxiety and stress [15]. However, there is little literature regarding coping strategies employed by MBC women in China.

Furthermore, there is a scarcity of data on the association between distinct coping techniques and emotional suffering among Chinese MBC women. Therefore, how they cope with the disease could highly impact their psychological well-being. In addition, how do women cope with an incurable disease in the light of social values may be different? For example, Chinese women with eastern values may cope with the disease differently from Western people. This study’s findings can help healthcare professionals and policymakers implement depression, anxiety, and stress reduction programmes, as well as essential measures to address the psychological needs and coping skills of women with MBC. This study aimed to assess depression, anxiety and stress among metastatic breast cancer (MBC) patients undergoing chemotherapy (CT) in Beijing, China. The purpose of this study will enable us to identify MBC patients at higher risk of depression, stress and anxiety. As a result, medical and mental health support can be directed to MBC patients at higher risk to control their psychological problems and improve their maladaptive coping.

## Methods

### Ethical considerations

The study was approved by the Human Research Ethics Committee Beijing Cancer Hospital (Reference Number: 2018KT106).

### Study design and setting

A cross-sectional study design involved MBC patients who attended follow-up treatment at the oncology unit of Beijing Cancer Hospital, China. Beijing Cancer Hospital is one of the largest hospitals in China. The study was conducted between May and December 2019.

### Study population and sampling

The study population was recruited through purposive sampling from one Cancer Hospital in Beijing. Purposive sampling is a non-probability method that can be applied to quantitative research. Purposive sampling (also known as judgment, selective or subjective sampling) is a technique in which researchers use their discretion in selecting members of the population to participate in the study. The reason is purposive sampling is used to better match the sample to the research’s goals and objectives, assisting researchers in answering the research question. Therefore, using this method, the researchers can choose participants based on how well they fit a specific profile, such as MBC patients on CT. Participants diagnosed with MBC and undergoing at least the first cycle of CT, with relapsed BC and aged 18 to 70 years above were included in this study. Participants were excluded if diagnosed with mental health illnesses such as depression or anxiety or who were taking prescription drugs, had severe comorbidity, and had apparent cognitive impairment. The Mini-Mental State Examination (MMSE) was used to determine cognitive impairment based on a 23/24 cut-off score [17]. Karnosky’s Performance Status (KPS) of less than 50 was used to assess participants’ functional impairment and health status [18]. Participants with a KPS of 50 or fewer or who were terminally ill were excluded due to the possibility of worsening symptoms. Estimation of sample size was based on the results of the previous study [19] using the Power and Sample (PS) size calculation software version 3.0.43, the desired significance level (α) 0.05, the population SD, (σ = 17.23), the difference in population means, (δ = 5.16), the power of the study (β) 80% and the ratio (m = 1), giving a total sample, n, of 176. A pilot study was carried out with 18 participants to establish the validity and reliability of the instruments and to assess the participant’s ability to understand the questions asked accurately. The first researcher administered the questionnaire to all those selected for the study.

### Measures

A self-administered survey questionnaire consisting of three sections was administered, which participants were asked to complete in 10 to 15 min. The first section collected data on socio-demographics, including age, marital status, level of education, household income, self-assessment of marriage quality, and medical insurance status. Medical variables include MBC diagnosis duration, metastasis site, pathologic type (estrogen receptor, progesterone receptor, and human epidermal growth factor receptor 2), CT treatment lines, and receiving single or combined treatment. The second section measures depression, anxiety, and stress. The measurement tool was the Chinese Depression Anxiety Stress Scale Version-21 (DASS-21) version [20, 21]. The Chinese version of DASS-21 has been shown to have good reliability in the Chinese population, including BC. DASS-21 questionnaire was a self-report measure of anxiety, depression, and stress, widely used to measure negative affection. There are seven items in each of the three sub-scales: Depression (DASS21-D), Anxiety (DASS21-A), and Stress (DASS21-S). Each item had a statement and four short response alternatives to express the severity, and it was scored from 0 (did not apply to me at all) to 3 (very severe) (Applied to me very much, or most of the time). The sum scores are calculated by adding the scores from each (sub) scale item and multiplying them by a factor of two. The cut-off points for depression were 0–9 (normal), 10–13 (mild), 14–20 (moderate), 21–27 (severe) and ≥ 28 (extremely severe). The cut-off points for anxiety were 0–7 (normal), 8–9 (mild), 10–14 (moderate), 15–19 (severe) and ≥ 20 (extremely severe). The cut-off points for stress were 0–14 (normal), 15–18 (mild), 19–25 (moderate), 26–33 (severe) and ≥ 34 (extremely severe) [22]. Cronbach’s alpha ranges from 0.76 to 0.84 for the original DASS’s psychometric properties. In contrast, the internal consistency ranges from 0.83 to 0.91 [21]. In this study, Cronbach’s coefficient (reliability) of the DASS-21 Chinese version was considered high for all subscales (0.90 for depression, 0.78 for anxiety, and 0.86 for stress). Thus, the DASS-21 Chinese version is a reliable and valid instrument that could be used for the Chinese population based on its acceptable internal consistency.

The third section measured was the Chinese version of Brief COPE. Brief COPE is an abbreviated version of the Cope Inventory and measures coping strategies. It has 28 items and measures 14 conceptually different coping strategies: self-distraction, active coping, denial, substance use, emotional support, instrumental support, behavioural disengagement, venting, positive reframing, planning, humour, acceptance, religion, and self-blame. Cronbach’s alpha for the subscales ranged from 0.50 (venting) to 0.90 [23]. The Brief cope inventory has been used to measure the coping strategies in Chinese women after hysterectomy surgery and breast cancer. The reported Brief Cope Inventory reliability was 0.71 and 0.79 [24]. Cronbach’s alpha was determined for each subscale in this study to measure internal consistency, and the value was 0.90, indicating strong internal consistency.

The data were analyzed using Statistical Package for Social Sciences Software (SPSS), version 26.0. Frequency distributions for each question were checked for outliers to identify incorrectly coded data. Cronbach’s alpha (α) coefficient was used to assess internal consistency as a measure of scale reliability. Descriptive statistics were used to calculate frequencies, percentages, measures of central tendency and socio-demographic data and survey subscales, mean and standard deviation (SD) for coping styles. Pearson’s correlation coefficient was used to check the correlation between MBC women’s coping styles and psychological distress. Finally, General Linear Regression (GLR) was conducted to explore the association of socio-demographic, clinical characteristics and coping styles with psychological distress.

## Results

A total of 176 MBC patients were involved in this study. The socio-demographic and clinical characteristics of the participants are shown in Table 1. Most participants were over 40 years old (*n* = 156, 88.6%), aged from 28 to 70 years old, with a mean age of 51.70 ± 9.25. Most of these MBC patients were married or cohabiting (*n* = 157, 89.2%) and were covered by health insurance (*n* = 163, 92.6%). In addition, 46% of the MBC patients in this study had been diagnosed within six months. The majority of them have bone metastasis (54.5%). 90 (51.1%) were receiving the first treatment, and 93(52.8%) MBC women were using single chemotherapy (CT) regimen rather than a combined CT regimen.

**Table 1:** Social-demographic and clinical characteristics of MBC women (*n* = 176)

Characteristics	n (%)
Age	
20–29	1( 0.6)
30–39	19(10.8)
40–49	52(29.5)
50–59	63(35.8)
60–70	41(23.3)
Marital status	
Single, divorced or widowed	19(10.8%)
Married or cohabiting	157(89.2%)
Education	
No formal education or primary school	19(10.8%)
Secondary school	49(27.8%)
High school/Technical secondary school	52(29.5%)
Junior college	30(26.0%)
University or higher	26(14.8%)
Monthly income (CNY)	
≤ 1500	62(35.2%)
1501–3000	77(43.8%)
3001–5000	19(10.8%)
≥ 5000	18(10.2%)
Medical payment method	
Self	13( 7.4%)
Health insurance	155(88.1%)
Public pay method	8( 4.5%)
Duration of MBC diagnosis	
≤6 months	81(46.0%)
6–12 months	25(14.2%)
12.01-24 months	26(14.8%)
≥ 24 months	44(25.0%)
Bone metastasis	
Yes	96(54.5%)
No	80(45.5%)
Lung metastasis	
Yes	64(36.4%)
No	112(63.6%)
Liver metastasis	
Yes	84(47.7%)
No	92(52.3%)
Brain metastasis	
Yes	15( 8.5%)
No	161(91.5%)
Treatment lines	
1st	90(51.1%)
2nd	42(23.9%)
≥ 3rd	44(25.0%)
Single or combined regimen	
Single	93(52.8%)
Combined	83(47.2%)

CNY (Chinese Yuan Renminbi): 1 Chinese Yuan equals 0.16 United States Dollar (Conversion last updated Feb 20, 2022, 12:02 UTC)

**Fig. 1:**
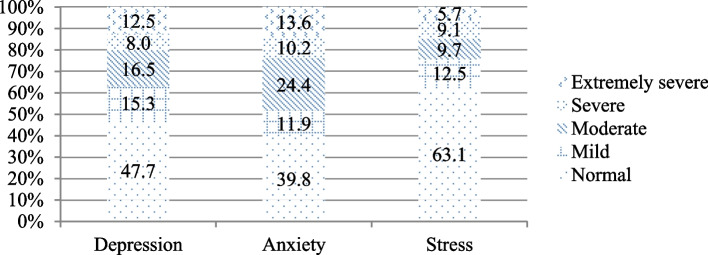
The incidence of MBC women’s Depression, Anxiety and Stress

Mean scores and standard deviations for depression, anxiety and stress are 12.07 ± 10.69, 10.64 ± 8.06 and 13.49 ± 9.97, respectively. Figure 1 shows the extent of depression, anxiety and stress. 63.6% of MBC women represented depression (mild 15.5%, moderate 16.5%, severe depression 8% & extremely severe 12.5%), while anxiety was estimated at 60.2% (mild 11.9%, moderate 24.4%, severe 10.2% & extremely severe 13.6%). The stress estimation was noted at 36.9% (mild stress 12.5%, moderate 9.7%, 9.1% severe stress & extremely severe 5.7%).

### Correlation between coping styles and psychological distress among MBC women

Table 2 shows the correlation between coping styles and psychological distress among MBC women. There was a positive correlation between self-blame, denial, behavioural disengagement, venting and depression (*r* = 0.442, 0.293, 0.260, 0.203, respectively) and a weak negative correlation between positive reframing and depression (*r* = -0.251). Positive correlations were found between behavioural disengagement, self-blame, denial, venting, religious coping, planning and anxiety; the coefficients were 0.415, 0.360, 0.329, 0.236, 0.188, and 0.149, respectively. Self-blame, denial, behavioural disengagement, venting, and self-distraction positively correlated with stress (*r* = 0.564, 0.397, 0.338, 0.269, 0.163, respectively). MBC patients used acceptance, planning, active coping, and positive reframing more frequently than disengagement coping strategies such as substance abuse, behavioural disengagement, denial, self-blame, and venting.

**Table 2:** Correlations between MBCwomen’s depression, anxiety, stress and coping styles (*n* = 176)

	Mean	Std. Deviation	Depression(*r*)	Anxiety(*r*)	Stress(*r*)
Acceptance	4.91	1.511	-0.106	0.066	0.018
Planning	4.52	1.534	-0.064	0.149^b^	0.072
Active Coping	4.44	1.476	-0.021	0.106	0.097
Positive Reframing	4.40	1.512	-0.251^b^	-0.022	-0.074
Self-distraction	4.29	1.415	0.051	0.144	0.163^a^
Use of Emotional Support	4.26	1.507	-0.005	0.122	0.099
Humour	4.24	1.586	-0.174	0.008	-0.073
Venting	4.21	1.351	0.203^b^	0.236^b^	0.269^b^
Self-blame	4.14	1.487	0.442^b^	0.360^b^	0.564^b^
Use of Instrumental Support	4.09	1.305	0.080	0.130	0.148
Denial	3.94	1.541	0.293^b^	0.329^b^	0.397^b^
Religion Coping	3.24	1.601	0.113	0.188^b^	0.142
Behavioural Disengagement	3.13	1.146	0.260^b^	0.415^b^	0.338^b^
Substance Use	2.15	0.487	0.042	0.140	0.024

^a^Correlation is significant at the 0.05 level

^b^Correlation is significant at the 0.01 level

Age, working status (employment), income, marital quality, bone metastasis, brain metastasis, number of metastatic sites, single or combination CT regimen, and CT lines match up to the general linear regression (GLR) analysis (*p*-value 0.25) to investigate depression prediction. In addition, the coping styles that were linked to depression were chosen. In general linear regression, poor marriage quality, venting, self-blame, and behavioural disengagement were predictors of depression. In contrast, positive reframing was a negative predictor of depression. The variance of determination R^2^ was 35.6%, as indicated in Table 3.

**Table 3:** General linear regression analysis examining the contribution of demographic/clinical variables and coping styles to the prediction of depression (*n* = 176)

Variables	SLR		GLR		
	*Crude b*(95%CI)	*P*-value	Adjusted *b*(95%CI)	*t* statistics	*P-*value
Age	0.102(-0.070,0.274)	0.243			
Marital status					
Single/Divorced/ Widowed/	1.340(-3.794,6.473)	0.607			
Married/Cohabiting	0				
Education level					
Low education	0.943(-2.328,4.215)	0.570			
High education	0				
Working status					
Employed	-2.510(-6.487,1.466)	0.214			
Unemployed	1.209(-2.089,4.508)	0.470			
Retired	0.482(-2.736,3.700)	0.768			
Income					
Low income	1.932(-1.329,5.194)	0.244			
High income	0				
Marriage quality					
Good	0				
Poor	5.615(1.860,9.370)	0.004	3.746(0.475,7.018)	2.262	0.025
Metastasis site					
Bone	-4.092(-7.235,-0.949)	0.011			
Lung	-0.893(-4.204,2.491)	0.595			
Liver	0.143(-3.049,3.335)	0.930			
Brain	-0.075(-5.785,5.635)	0.979			
Number of Metastasis sites	-1.035(-2.3957,0.326)	0.135			
Duration of Metastasis	0.014(-0.047,0.075)	0.655			
CT regimen					
Single	-3.230(-6.387,-0.073)	0.045			
Combine					
CT lines					
1st	0				
≥2nd	2.868(-0.292,6.029)	0.075			
Coping styles					
Positive Reframing			-2.862(-3.800,-1.923)	-6.024	< 0.001
Venting			1.149(0.060,2.238)	2.084	0.039
Self-blame			3.143(1.175,3.112)	4.371	< 0.001
Denial					
Behavioral disengagement			2.215(0.910,3.521)	3.352	0.001

*R*^*2*^ = 0.356

Stepwise multiple linear regression method applied

Simple linear regression (SLR) tests indicated that marital status conformed to the multiple linear regression analysis (*p*-value < 0.25) to explore the prediction of anxiety. In addition, the coping styles which correlated to anxiety were selected. According to the results from GLR, poor marriage quality, self-blame, and behavioural disengagement were predictors of anxiety. All those predictors explained a 25.2% variance of anxiety in MBC patients, the specifics of these results are in Table 4.

**Table 4:** GLR analysis examining the contribution of socio-demographic/clinical variables and coping styles to the prediction of anxiety (*n* = 176)

Variables	SLR		GLR		
	*Crude b*(95%CI)	*P*-value	Adjusted *b*(95%CI)	*t* statistics	*P-*value
Age	-0.030(-0.160,0.101)	0.655			
Marital status					
Single/ Divorced/ Widowed/	3.299(-0.545,7.142)	0.092	3.714(0.335,7.092)	2.170	0.031
Married/ Cohabiting					
Education level					
Low education	-0.654(-3.121,1.814)	-0.602			
High education	0				
Working status					
Employed	-0.438(-3.450,2.574)	0.775			
Unemployed	-0.814(-3.303,1.675)	0.519			
Retired	1.057(-1.366,3.479)	0.390			
Income					
Low income	-0.161(-2.631,2.308)	0.898			
High income	0				
Marriage quality					
Good	0				
Poor	1.198(-1.640,4.036)	0.406			
Metastasis site					
Bone	-0.667(-3.080,1.746)	0.568			
Lung	-0.214(-2.714,2.285)	0.866			
Liver	0.923(-1.480,-3.327)	0.449			
Brain	1.199(-3.104,5.502)	0.583			
Number of metastasis sites	0.057(-0.967,1.089)	0.914			
Duration of metastasis	0.013(-0.034,0.059)	0.588			
CT regimen					
Single	-1.022(-3.426,1.382)	0.403			
Combine					
CT lines					
1st	0				
≥2nd	0.848(-1.555,3.250)	0.487			
Coping styles					
Planning					
Venting					
Self-blame			1.311(0.594,2.028)	3.609	< 0.001
Denial					
Religion coping					
Behavioral disengagement			2.426(1.461,3.391)	4.962	< 0.001

*R*^*2*^ = 0.252

Stepwise multiple linear regression method applied

Poor marriage quality, bone metastasis, lung metastasis, the number of metastasis sites and single or combined chemotherapy regimen were measured up the general linear regression analysis (*p*-value < 0.25) to explore stress prediction. In addition, the coping styles which correlated to stress were selected. According to multiple linear regression (MLR) results, poor marriage quality, self-blame, denial, and behavioral disengagement were predictors of stress. All those predictors accounted for a 35.4% stress variance in MBC patients, and the detailed results are given in Table 5.

**Table 5:** GLR analysis examining the contribution of socio-demographic/clinical variables and coping styles to the prediction of stress (*n* = 176)

Variables	SLR		GLR		
	*Crude b*(95%CI)	*P-*value	Adjusted *b*(95%CI)	*t* statistics	*P-*value
Age	-0.015(-0.177,0.147)	0.854			
Marital status					
Single/ Divorced/Widowed	0.449(-4.463,5.362)	0.857			
Married/Cohabiting					
Education level					
Low education	-0.763(-3.831,2.305)	0.624			
High education	0				
Working status					
Employed	0.071(-3.660,3.803)	0.970			
Unemployed	0.231(-2.858,3.319)	0.883			
Retired	-0.267(-3.282,2.749)	0.862			
Income					
Low income	1.083(-1.975,4.141)	0.485			
High income	0				
Marriage quality					
Good	0				
Poor	5.307(1.793,8.821)	0.003	3.964(0.890,7.039)	2.547	0.012
Metastasis site					
Bone	-1.999(-4.983,0.985)	0.188			
Lung	-2.073(-5.167,1.020)	0.188			
Liver	0.348(-2.639,3.335)	0.818			
Brain	-1.917(-7.240,3.407)	0.478			
Number of metastasis sites	-0.782(-2.055,0.491)	0.227			
Duration of metastasis	-0.024(-0.083,0.034)	0.414			
CT regimen					
Single	-3.255(-6.205,-0.304)	0.031			
Combined					
CT lines					
1st	0				
≥2nd	1.046(-1.936,4.028)	0.490			
Coping styles					
Self-distraction					
Venting					
Self-blame			2.446(1.497,3.394)	5.093	< 0.001
Denial			1.040(0.002,2.078)	1.980	0.049
Behavioral disengagement			1.511(0.264,2.757)	2.394	0.018

*R*^*2*^ = 0.354

Stepwise multiple linear regression method applied

## Discussion

This study revealed that 52.3% of Chinese MBC women suffered from depression, 60.2% had anxiety, and 36.9% experienced stress. Our findings outperformed those of another study that examined psychological distress and quality of life in Chinese early-stage breast cancer patients undergoing CT in China (44.1% depression and 35.2% anxiety) [24]. Furthermore, using the Distress Thermometer, 61% of MBC women reached the cut-off for psychological distress, comparable to our finding [25]. In any case, MBC women’s anguish plays an essential part in their disease trajectories and deserves further consideration. As a result, screening for psychological discomfort should be done regularly.

Coping strategies in cancer patients are pivotal in lowering psychological distress (depression, anxiety and stress). General linear regression analyses revealed poor marriage quality, self-blame, and behavioural disengagement were predictors of anxiety among MBC women. Our findings were consistent with those of Donovan-Kicken et al., who discovered that cancer patients who used coping techniques such as self-blame had greater levels of depression and anxiety [26]. It was anticipated that the MBC women felt they did not manage their health well or that economic challenges resulted from large sums of medical costs for cancer treatment. The chance of extending life can be increased. However, this situation increases the burden on more challenging financial expectations around medical interventions and intensive treatment. Another explanation for the self-blame could be that the disease had become a burden on their family. As a result, the family structure had to be reorganized, especially for the younger MBC patients. A possible explanation for psychological distress is the difficulties associated with the responsibility for caring for or raising their children for the next generation.

Denial was one of the coping strategies associated with psychological distress (depression, anxiety, and stress) among MBC women in this study. Denial contributed to a higher degree of psychological distress, such as depression and anxiety [12]. The diagnosis may threaten or harm the MBC patients. The denial coping may spend energy on avoiding evidence to the contrary, and as a predictor, could put them into the stress status. Maintaining inside equilibrium may call for denial at the beginning of MBC diagnosis. The acute distress during the vulnerable new MBC diagnosis period could be shielded by the transitorily escaping or denial. Nonetheless, denial coping may help MBC patients sustain a body-mind balance temporally; however, it should not exist among MBC women in the long run. It could drive them into a stressful mental state.

This study highlights that behavioral disengagement is one of the coping strategies identified. The coping style reflects MBC women’s tendency to give in or reduce their efforts in difficult situations. This finding is consistent with a previous study that behavioral disengagement is associated with psychological well-being [27]. Therefore, the plausible explanation could be that people using behavioral disengagement may lose control of the stressful event, making them feel worse and more depressed. It is possible to conclude that Chinese MBC women who choose behavioural disengagement coping may lose coherence to life activity and abandon dealing with concerns in their everyday lives. Therefore, encouraging MBC women to renormalize their lives and contribute to their health problems and daily activities could benefit them long-term.

The findings of our study revealed that poor marriage quality is associated with coping styles for the prediction of depression. Participants’ bond with their partner is frequently their key coping resource, providing timely, strong support for married women [28]. As a result, women with MBC use different mechanisms to cope with dramatic changes in all aspects of their lives [29]. Poor marital adjustment was significantly associated with cancer patients’ psychological well-being [30]. The relationship between marital quality status and cancer has been evaluated in several studies [31, 32], with findings strongly suggesting that being in a positive marriage is a prognostic factor of better survival concerning all-cause mortality and cancer-specific mortality death. Social support improves and influences psychological well-being through certain coping mechanisms [33]. There is evidence that social support aided cognitive processing by minimizing intrusive rumination and avoidance, lowering psychological discomfort, giving women the strength to persevere, and assisting them in accepting and coping with the constraints of MBC.

For the CT regimen, a single CT regimen was associated with coping styles to predict depression. It is well documented that cancer and its treatment are linked to alarming psychological disorders, which may vary depending on the types and combinations of therapy, dosages, and the number of treatment cycles [34]. However, the causal association between single and combined regimens cannot be ascertained due to the cross-sectional nature of this study. Therefore, longitudinal studies are warranted to examine the directionality of these associations in more detail.

The present finding suggests that positive reframing coping protects MBC women from depression. Positive reframing of a negative encounter is a coping mechanism linked to depression, with positive reappraisal linked to reduced depressive symptoms [35]. The researcher suggested positive thinking can be used as a bulwark against hopelessness in life-threatening illnesses. Positive reframing enhances MBC women’s coping efforts through a strong sense of control and confidence as an adaptive coping style. Therefore, positive reframing coping as an efficient coping strategy may provide ongoing motivation for life and daily activities, which MBC women can exercise to prevent depressive symptoms.

Our hypothesis regarding the association between venting and depression moderators was partially supported and consistent, with venting coping associated with depressive symptoms [36]. Hence, this might be explained by MBC women who used vented coping were unconfident in their ability to deal with the disease and saw their current situation as out of control, resulting in despair mediated by catharsis. Furthermore, our study showed that interpersonal accountability and openness might reduce the likelihood of choosing and utilising venting coping behaviours like becoming angry with others, losing one’s temper, or blowing up in front of others [37]. Thus, enhancing MBC patients’ interpersonal communication skills to limit the need for venting coping may lessen depression symptoms in the future.

### Limitations

There are some limitations to this study. First, this cross-sectional study could not draw any causal association between tested variables and MBC. Therefore, longitudinal studies are needed to identify the causal association further and monitor the trajectory of MBC. Second, as with other non-probability sampling techniques, purposive sampling is prone to bias. Third, the study was conducted at one urban hospital in China; therefore, the generalization of these results is difficult. Sample drawn from several hospitals across the country is needed for better generalizability.

### Implications for practice and research

The findings should spur further intervention research to link BC and treatment in a more adaptable way. Furthermore, the evidence-based coping skills taught may need to be adapted to accommodate MBC patients’ psychological well-being. In addition, an increased understanding of coping methods among healthcare professionals might benefit MBC patients at risk of psychological discomfort. Finally, the findings have ramifications for developing psychological interventions to reduce self-blame coping, behavioral disengagement, denial, and venting.

## Conclusion

Depression, anxiety, and stress prevalence are high in MBC women. Therefore, assessment of psychological distress (depression, anxiety, and stress) is important to recognize MBC patients who need help and further medical and mental help support. Furthermore, this study’s findings can increasingly highlight that depression, anxiety, and stress are substantial problems in MBC patients. Given the increasing MBC women’s psychological distress, interventions are needed to reduce depression, anxiety, and stress.

## Data Availability

The datasets used and analysed during the current study are available upon reasonable request from the corresponding author.
